# Sickle Cell Disease and Venous Thromboembolism

**DOI:** 10.4084/MJHID.2011.024

**Published:** 2011-05-24

**Authors:** Zohreh Rahimi, Abbas Parsian

**Affiliations:** 1 Medical Biology Research Center, Kermanshah University of Medical Sciences, Kermanshah, Iran; 2 Department of Biochemistry, Medical School, Kermanshah University of Medical Sciences, Kermanshah, Iran; 3 Division of Neuroscience & Behavior, NIAAA, National Institutes of Health, Rockville, Maryland, USA

## Abstract

Hemoglobin S in homozygous state or in combination with one of the structural variants of Hb D-Punjab, Hb O-Arab, Hb C or β-thalassemia mutation results in sickle cell disease (SCD) that is characterized by chronic hemolytic anemia and tissue injury secondary to vasooclusion. A chronic hypercoagulable state in SCD has been established with the increased risk of thromboembolic complications in these patients. The goal of present review is to survey of the literature related to thromboembolic events and genetic risk factors involved in the manifestation of these events in SCD patients with focus on studies from Mediterranean countries. Also, this review covers the pathogenesis of hypercoagulability and alteration in the components of hemostasis system.

## Introduction:

The Hb S that results from substitution of valine for glutamic acid at position 6 of β-globin chain was the first hemoglobin variant to be discovered.^1^ This abnormal hemoglobin is the most clinically important structural variant of hemoglobin with highest frequency in Africa, Saudi Arabia, and India.^1^,^2^ Sickle cell disease (SCD) results from the homozygous state of the mutation, or a compound heterozygous state with one of structural variants of Hb D-Punjab, Hb O-Arab, Hb C or β-thalassemia mutation in the other β-gene.^2^ This group of disorders characterized by the polymerization of deoxygenated hemoglobin S into rigid rodlike polymers, causing the sickling of the erythrocyte.^3^

The β^S^ mutation has been found to be in linkage disequilibrium, with five distinct, typical (common) β-globin gene cluster haplotypes. Four of these haplotypes are known as African haplotypes (Bantu, Benin, Senegal, and Cameroon), the fifth is the Arab-Indian haplotype, which was originally described in the eastern oases of Saudi Arabia and among the tribal Adivasi population of India.^4^ The clinical severity and hematological manifestations of sickle cell anemia are varied and are influenced by other pleiotropic effects of the haplotype background linked to the β^S^ gene.^5^,^6^

SCD is known as a hypercoagulable state in which various hemostatic systems both in steady state and during vaso-occlusion are perturbed with increased activation of the coagulation system and platelets, thrombin generation, and occurrence of thrombosis.^7^–^10^

## Cerebral venous and sinus thrombosis (CVST):

The most important cause of stroke in SCD patients is large-vessel cerebral arterial complication that might be the result of abnormal adhesive and procoagulant properties of RBC, which produce endothelial damage, secondary intimal proliferation, and thrombosis.^10^–^12^ The incidence of cerebro-vascular complications in SCD is 5 to 10% (13). In 11% of sickle cell anemia patients stroke is occurred by age 20, with infarction mainly in the internal carotid and middle cerebral arteries.^14^

Association between SCD and cerebral thromboembolic events has been investigated in several studies. Alli et al^15^ reported the presence of skull bone infarction crisis and deep vein thrombosis in a boy with homozygous sickle cell anemia (SCA). Also, in individuals with sickle cell trait (SCT) cerebral infarction and stroke is not rare and there are several case reports related to the incidence of stroke in these individuals.^16^–^19^ There are few studies from Mediterranean countries reported the occurrence of CVST and stroke in SCD patients. Sidani et al^20^ have reported the existence of venous and sinus thrombosis and stroke in a Lebanese young homozygous sickle cell patient. However, in this patient the presence of proteins C, S, antithrombin III, factor V Leiden (FVL), and prothrombin mutations as well as anticardiolipin antibodies were negative.^20^ Also, a 25-year-old French SCD patient with cerebral vein thrombosis and bilateral thalamic infarcts has been reported.^21^ Further, the report of Ozu et al^22^ indicated the occurrence of sinus thrombosis and thalamic infarcts in a 2-year-old child from Turkey. In addition, Radhakrishnan et al^16^ reported the presence of stroke in two young adult sickle cell trait individuals from Libya.

## Pulmonary embolism and deep vein thrombosis:

A link between hypercoagulability in SCD patients and pulmonary hypertension (PHT), increased pulmonary artery pressure and pulmonary vascular resistance, has been established.^7^,^23^ Pulmonary hypertension occurs in approximately 30% of adult patients with SCD and is a risk factor for early death.^24^ In studies using echocardiograms of patients with SCA who were examined at referral centers the prevalence of PHT was 20 to 30%. In autopsy studies, approximately 75% of patients with SCA present histological evidence of PHT.^25^

Hemolysis has been identified as one of the potential driving forces of the hypercoagulable state and developing PHT in SCD.^23^,^26^ However, Beers et al^27^ did not detect a significant difference in haemolysis between patients with and without PHT and did not find a direct link of the hypercoagulable state to mild SCD related PHT.

A mechanism related to nitric oxide (NO) scavenging by free hemoglobin has been implicated in the pulmonary arterial disease of sickle cell anemia. Hemolytic anemia through hemolysis and release of hemoglobin into plasma which consumes NO and release arginase into plasma leads to resistance to NO-dependent vasodilatory effects. This is an important process in the development of PHT and shortened life expectancy in patients with SCD.^25^,^28^ Bunn et al^29^ reviewed the literature related to the influence of NO depletion by plasma hemoglobin in the microcirculation on the pathogenesis of PHT in SCD patients. They concluded that pulmonary hypertension per se was not a major cause of death in sickle cell patients and therapies that might enhance the availability of NO to the vasculature have thus far been ineffective and/or toxic.

In a large case-control study on 515 hospitalized black patients with thromboembolism and Hb S and C and 555 black controls revealed that persons with SCT experienced approximately a 2-fold increased risk of venous thromboembolism (VTE) compared with persons with the wild-type genotype, Hb AA. This increased risk was observed both for idiopathic and provoked VTE as well as for first and recurrent VTE. In addition, pulmonary embolism (PE) risk (without DVT) was significantly increased (approximately 4-fold) among those with SCT, whereas the risk of a DVT (without PE) was not significantly increased.^30^ The increased risk of VTE is attributed to subclinical sickling of red cells.^30^

Results of autopsy of 12 SCD patients suggested that pulmonary thromboemboli may be a late complication of PHT and an in situ thrombotic arteriopathy underlies the development of PHT in most patients with sickle cell disease.^29^ Small vessel thrombosis is one of underlying cause of pulmonary hypertension (PHT) among patients with SCD (31) and an association between thromboembolism and PHT has been suggested.^32^ Staser et al^33^ reported a rare phenomenon of calcified pulmonary thromboembolism in an African American boy with SCD.

Higher prevalence of pulmonary embolism in African American SCD patients below 40 years of age (0.44%) compared to non SCD African Americans (0.12%) has been reported.^34^ Clinical assessment and/or autopsy findings at the time of death among 141 adults with sickle cell disease (SCD) over a 25-year period revealed that PHT is the leading cause of death (26.2%) and thromboembolism was the fifth symptom leading to death in these patients. In another study with the goal of determining the most common pathologic findings in autopsy cases with sickle cell lung disease indicated the high percentage of PHT (33.3%) in these autopsy samples.^35^ The risk of thromboembolic complications in SCD patients appears to be higher following splenectomy.^7^,^36^

## Genetic of Thromboembolism:

Venous thromboembolism is believed to be caused by genetic and acquired risk factors. The inherited hypercoagulable syndromes primarily affect veins, and only rarely cause arterial thrombosis. The acquired hypercoagulable states, such as the antiphospholipid antibody syndrome, are more commonly implicated in arterial stroke.^37^

The role of inherited thrombophilia in the pathogenesis of sickle cell thromboses has been reported in few studies. In these studies the frequency of thrombophilic mutations of FVL, prothrombin G20210A, and methylenetetrahydrofolate reductase (MTHFR) C677T and their association with incidence, and recurrence of thromboembolism among SCD patients have been examined.

The factor V Leiden mutation is caused by a single point mutation at nucleotide 1691, leading to an Arg/Gln amino acid exchange at this position is the most frequent inherited risk factor for thromboembolism in Caucasians with a prevalence of 5% in the general population.^13^ Higher frequency of this mutation in β-thalassemia patients and its association with deep venous thrombosis and cerebral venous and sinus thrombosis has been reported.^38^–^40^

There are many studies reported the low frequency of thrombophilic mutations in SCD patients and the lack of association between these mutations and risk of thromboembolism. Helley et al^13^ in a large sample of SCD patients from Africa indicated the absence of FVL mutation in these patients. However, the gene frequency of the FVL in black SCD subjects from West Indies and Maghrib were 2.5 and 1.1%, respectively. They concluded that in sub-Saharan African SCD patients FVL is not an additional risk factor for thrombosis.^13^ Also, In African American with SCD this mutation was uncommon and was not found to be responsible for stroke in these patients.^41^,^42^ Further, in the study of Wright et al^43^ among SCD patients from Jamaica no FVL mutation was found in these patients. However, a significant reduction in the median activated protein C resistance ratio compared to controls was observed in their study. Moreira Neto et al^44^ reported no prothrombin G20210A mutation in SCD patients from Brazil. The frequency of heterozygous FVL mutation was 1.8% in their studied patients. In another study from Brazil, Andrade et al^45^ studied the prevalence of the FVL mutation, MTHFR C677T polymorphism, and prothrombin gene variant in SCD patients. It was reported no significant difference between SCD patients and the control population for the prevalence of the studied thrombophilic mutations. However, among nine studied SCD patients with vascular complications of stroke or deep vein thrombosis they found only one patient to be a carrier for FVL. Also, Kordes et al^46^ reported no medical history of deep vein thrombosis in two SCA patients homozygous for FVL mutation from Iraq. Fawaz et al^47^ have compared the prevalence of FVL mutation and prothrombin gene variant in SCD patients and controls from Eastern Saudi Arabia. Their study revealed that there is no association between the mutations and SCD. Furthermore, Zimmerman et al^48^ reported no association between thrombophilic mutations of MTHFR C677T and platelet glycoprotein IIIa (GPIIIa) C1567T with SCD.

In contrast, in spite of the moderate prevalence of FVL mutation (2.97–5.5%) among normal population of Iran^49^,^50^ a high prevalence of FVL mutation (14.3%) among Iranian SCA patients^9^ has been found with a significant association between this mutation and SCA with odds ratios (OR) of 6.5 (95% confidence intervals [CI] 1.19–35.33, p = 0.03). In addition, increased prevalence of the FVL mutation in SCT individuals and sickle/β-thalassemia patients was not statistically different from controls (OR=3.84, 95% CI 0.49–29.9, p = 0.19 and OR=3.77, 95% CI 0.31– 45.9, p = 0.29, respectively).^9^ Also, in a study from Brazil it was suggested that MTHFR C677T might be a risk factor for vascular complications in SCD.^44^

According to the literature, there have been three studies of inherited risk factors of venous thromboembolism in SCD patients from Southern Mediterranean countries^51^–^53^ which report high prevalence of thrombophilic mutations in SCD patients and their association with thromboembolism in these patients. Among Lebanese sickle/β^0^-thalassemia patients, a high prevalence of thrombophilic mutations of FVL (42%), homozygous and heterozygous MTHFR C677T (59%), and prothrombin G20210A (8%) has been reported.^51^ In this report sickle-β-thalassemia patients were 5.24 and 4.39 times more likely to have FVL mutation as compared to the normal controls and thalassemia intermedia patients, respectively (p <0.05). Also, the presence of extensive large vessel thrombosis in a sickle/β^0^-thalassemia patient from Lebanon doubles heterozygous for FVL and MTHFR C677T (homozygous for FVL and heterozygous for MTHFR has been reported.^52^ Further, in a sickle cell anemia patient from Israel, Koren et al^53^ reported the recurrent of cerebrovascular accident and deep venous thrombosis. Activated protein C resistance due to FVL heterozygous and heterozygocity for the MTHFR C677T have been diagnosed and suspected to be the risk factors that contribute to the development of the deep vein thrombosis in this SCA patient.

## Pathogenesis of Hypercoagulability:

The pathogenesis of hypercoagulability is considered to be multifactorial. Altered components of hemostasis system in SCD have been suggested. Low plasma levels of protein C, protein S, and antithrombin III, elevated plasma levels of thrombin-antithrombin (TAT) complexes, prothrombin fragment 1+2 (F1+2), D-dimer complexes, and circulating antiphospholipid antibodies, platelet activation during vaso-occlusive crisis, abnormal external exposure of phosphatidylserine (PS) and adherence of sickle erythrocytes to the vascular endothelium, reducing NO level in the presence of hemolytic anemia, and increased tissue factor expression have been detected in SCD patients.^8^,^12^,^36^ These abnormalities of hemostatic system in SCD are leading to increase risk of thrombosis.

## Coagulation Factors and Inhibitors:

El-Hazmi et al^54^ reported significantly reduced levels of proteins C and S in SCD patients with the highest prevalence of deficiency in patients with a severe form of disease and frequent episodes of crisis. Lower levels of the naturally occurring anticoagulants protein S and protein C which are found in SCD patients could be attributed to either hemostatic abnormalities or hepatic dysfunction. Liesner et al^55^ reported that children with SCD have a reduction in levels of the majority of the coagulation inhibitors (protein C and S) and increased thrombin generation (thrombin-antithrombin complexes and prothrombin fragment 1+2 in the steady-state which is only partially reversed by transfusion. Onyemlukwe et al^56^ described significantly lower level of serum AT-III in patients with SCD compared to controls. Bayazit et al^57^ in a survey of SCA anemia patients in a steady state from Turkey found a significant lower level of protein C and AT levels in patients with SCA compared to controls. Also, they reported non significant lower levels of protein S in the patients than in the controls. They suggested that both hemostatic abnormalities and hepatic dysfunction contribute to low levels of natural coagulation inhibitors in SCA patients.

## Abnormal Structure of Red Blood Cells Membrane:

External exposure of phosphatidylserine has been implicated in the pathogenesis of adhesion of erythrocytes to endothelium in SCD. During deoxygenation, the amino phospholipids and phosphatidylethanolamine, are exposed to external membrane of red blood cells (RBC) and permanently fixed in irreversibly sickled cells leading to promote coagulation of red blood cells and platelets.^58^ The enzyme of scramblase in a Ca ^++^ dependent process which is modified by sulphydryl is involved in the translocation of PS from inner to outer layer of membrane.^59^ The increased oxidative stress in hemoglobinopathies^60^ increases sulphydryl modification and external exposure of PS. This process is a signal for RBC removal in apoptosis and a docking site for proteins involved in coagulation processes.^61^–^64^ Altered structure of RBC membrane in sickle cell disease due to external exposure of PS in the outer membrane increases adhesion of sickle erythrocytes to endothelial cells and microvascular occlusion. This alteration decreases red blood cell survival by increased splenic clearance of PS positive erythrocytes.^65^ Annexin V through calcium dependent mechanism binds to anionic phospholipids (primarily PS) exposed at external membrane layer. A high level of normal binding of annexin V to erythrocytes in sickle cell disease has been demonstrated. Painful vasoocclusive crises may partly result from these abnormal adhesive RBCs interacting with adhesion receptors on endothelia.^66^ Setty et al^67^ revealed a significant positive correlation between PS positive RBC and adhesion to vascular endothelium.

In SCD patients the presence of Arab-Indian and Senegal haplotype backgrounds of β^S^ gene compared to African haplotypes of Benin, Bantu and Cameroon are associated with higher levels of Hb F and amelioration of anemia.^4^,^5^ The benefit of Hb F effect in SCD patients has been shown that higher levels of F cells have a concomitant decrease in the numbers of microvesicles and PS-positive cells.^68^ The association between in vivo PS positive RBC and thrombin generation in SCD patients has been demonstrated by Setty et al.^69^ Westerman et al^70^ study indicated that posphatidylserine positive RBC are a significant source of PS (+) vesicles in SCA and the levels of circulating RBC-derived vesicles are increased in SCA. They reported a linkage between vesicle levels and activation of thrombin. The correlation between subpopulations of PS (+) erythrocytes and the risk of stroke in patients with SCD has also been demonstrated.^71^ Overall, the role of RBC in the coagulation activation has been shown that includes a significant correlation between the numbers of RBCs with external exposure of phosphatidylserine with plasma markers of thrombin generation, such as prothrombin fragment 1+2 (F1+2), D-dimer and plasmin- antiplasmin complexes. However, such association was not detected between phosphatidylserine-positive platelets and markers of thrombin generation.^68^,^69^

## Elevation of Tissue Factor Expression:

Tissue factor (TF) is a transmembrane glycoprotein that is involved in the activation of prothrombin and thrombin formation.^72^ Endothelial cells through expression of TF initiate coagulation. Significant elevation of TF in blood monocytes and circulating endothelial cells of SCD patients has been demonstrated that could be responsible for activation of the coagulation system in SCD patients.^72^,^73^

The mechanisms of activation TF are 1) increased heme levels through hemolysis which induces TF expression on the surface of endothelial cells,^36^ 2) ischemia-reperfusion injury (hypoxia/reoxygenation),^74^ 3) increased levels of soluble CD40 ligand through activation of platelets and exposure of CD40 ligand on their surface.^75^ In addition, the over expression of markers of endothelial activation such as endothelial adhesion proteins (intercellular adhesion molecule-1 [ICAM-1], E selectin [ELAM-1], P selectin and vascular cell adhesion molecule-1 [VCAM-1] has been reported in SCD.^76^

The utility of anticoagulant therapy as prophylaxis in hemolytic anemias such as SCD and novel approaches, including anti-hemolytic therapies, hemoglobin scavengers and NO donors have been suggested that could decrease the occurrence of thrombotic complications.^36^

## Conclusions:

There are several evidences that prove the multifactorial pathogenesis of hypercoagulability in SCD that include disturbing red blood cell phospholipids membrane asymmetry, abnormal activation of endothelial cells and other blood cells. The hypercoagulable state in SCD patients could be more complicated in the presence of inherited and acquired risk factors of thrombosis. Future studies on the influence of thrombotic risk factors on the incidence of thromboembolic events in SCD patients could elucidate the actual risk of thromboembolism in these patients.
